# Inflammation initiates a vicious cycle between obesity and nonalcoholic fatty liver disease

**DOI:** 10.1002/iid3.391

**Published:** 2020-12-17

**Authors:** Yunfei Luo, Hui Lin

**Affiliations:** ^1^ Department of Pathophysiology, Schools of Basic Sciences, Jiangxi Provincial Key Laboratory of Tumor Pathogens and Molecular Pathology Nanchang University Nanchang China

**Keywords:** adipose tissue inflammation, NAFLD, obesity, obesity‐induced inflammation

## Abstract

Low‐level of chronic inflammation activation is characteristic of obesity. Nonalcoholic fatty liver disease (NAFLD) is closely linked to obesity and is an emerging health problem, it originates from abnormal accumulation of triglycerides in the liver, and sometimes causes inflammatory reactions that could contribute to cirrhosis and liver cancer, thus its pathogenesis needs to be clarified for more treatment options. Once NAFLD is established, it contributes to systemic inflammation, the low‐grade inflammation is continuously maintained during NAFLD causing impaired resolution of inflammation in obesity, which subsequently exacerbates its severity. This study focuses on the effects of obesity‐induced inflammations, which are the underlying causes of the disease progression and development of more severe inflammatory and fibrotic stages. Understanding the relationship between obesity and NAFLD could help in establishing attractive therapeutic targets or diagnostic markers in obesity‐induced inflammation response and provides new approaches for the prevention and treatment of NAFLD in obesity.

## Graphical abstract

Obesity plays a vital role in the development of associated nonalcoholic fatty liver disease by affecting several inflammatory reactions via adipose tissue, vascular, intestinal, skeletal muscle, and brain, this process is associated with adipose tissue inflammation, inflammatory factors in the blood, intestinal inflammation, skeletal muscle inflammation, and brain tissue inflammation.

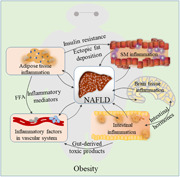

## 1 INTRODUCTION

Overweight and obesity are characterized by dysfunction or excessive accumulation of fat that may damage health. According to the latest data from the World Health Organization, between 1975 and 2016, the prevalence of obesity almost tripled, and at least 2.8 million people died of overweight or obesity each year. Obesity was once a problem that pertains to high‐income countries but is now widely in low and middle‐income countries as well.^1^ Obesity triggers diseases such as Type 2 diabetes (T2DM), hypertension, nonalcoholic fatty liver (NAFLD), hypogonadism, obstructive sleep apnea, asthma, and stress urinary incontinence.^2^ Concurrently, with progression of compromise to glucose uptake, insulin and leptin resistance, low‐grade inflammation, modified sympathetic activity accompanied by reduced noradrenergic innervations, and thermogenesis, obesity increased the risk of these long‐lasting energy balance disorders.^3^

**Figure 1:**
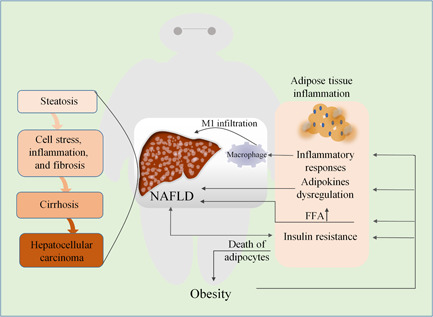
The relationship between obesity‐induced adipose tissue inflammation and NAFLD. Obesity changes the composition of immune cells in adipose tissue, thereby disrupting energy storage or consumption. This in turn triggers inflammation of adipose tissue, and the death of adipocytes further aggravating obesity. Inflammation of adipose tissue, resulting in secretion of factors (known as adipokines), increases generation of FFA and infiltration of M1 macrophages in the liver, as well as insulin resistance. Together, they influence the progression of NAFLD, leading to steatosis, liver inflammation and fibrosis, cirrhosis, and even increased risk of hepatocellular carcinoma. Systemic insulin resistance due to cirrhosis can exacerbate the inflammatory state of adipose tissue by reducing its energy storage capacity. FFA, free fatty acid; NAFLD, nonalcoholic fatty liver disease

As obesity progresses, it results in high prevalence and severity of NAFLD and even promotes of liver‐specific mortality among patients with NAFLD.^4^ NAFLD is intensified by obesity and is an emerging health problem that affects onethird of adults and more and many children in developed countries.^5^ It also reflects a series of related diseases and involves a range of pathological changes in the liver, starting with accumulation of triglycerides (TGs) in lipid droplets of hepatocytes, which is a causative factor in development of steatosis, and steatosis confirms the important connections between inflammation, cell death, and fibrosis, which is termed as nonalcoholic steatohepatitis (NASH), it can induce of cirrhosis, and elevate the risk of developing hepatocellular carcinoma^6^ (Figure 1). In addition, the difference between NASH and steatosis is that it shows the characteristics of inflammation, and markedly raises the risk of major liver disease in the future.^7^ Low‐level chronic inflammation and lipid accumulation in in some organs, especially the liver are considered the main triggers involved in the pathogenesis of NAFLD.^8^ Accumulating evidence suggests that related inflammation caused by obesity, including adipose tissue (AT) inflammation, inflammatory factors in the blood, intestinal inflammation, skeletal muscle inflammation, and brain inflammation may play a key role in the course of this disease. Furthermore, since the early stages of NAFLD usually have no obvious symptoms,^9^ the inflammation associated with obesity is of great significance for early identification and diagnosis of NAFLD.

Consequently, due to lack of approved medical interventions for NAFLD, alleviation of obesity would be beneficial to people's health and is a reasonable option to prioritize the treatment of NAFLD, as it can lower blood lipids, thus an improvement of the incidence of fatty liver.^10^ It is well known that the obesity is associated with chronic low‐grade inflammation in many tissues including liver, AT, vascular, intestinal, skeletal muscle, kidney, pancreas, and brain tissues,^11^, ^12^, ^13^, ^14^ which are associated with factors such as monocyte chemotactic protein (MCP‐1), CC chemokine receptor 2 (CCR2), tumor necrosis factor‐α (TNF‐α), toll‐like receptor 4 (TLR4), C‐Jun N‐terminal kinase (JNK), macrophages, and various types of T cells and B cells.^15^ Obesity leads to low‐level systemic inflammatory response, which in turn affects all aspects of the body, especially nonalcoholic fatty liver. This study focuses on obesity‐induced inflammations, especially the effects of fat inflammation, inflammatory factors in the blood, intestinal inflammation, and skeletal muscle inflammation on NAFLD, and reviews some mechanisms of interaction with each other.

### 1.1 The obesity‐driven pathophysiology of hepatic steatosis

The mechanism of progressive liver damage that worsens in NAFLD is characterized by a “multiple‐hit process.” The first “hit” contributes to the emergence of increased liver fat, while the subsequent multiple factors cause expansion of the inflammation.^16^ In the initial step, hepatic steatosis occurs through enlargement of the imbalance between TG acquisition and depletion, and obesity leads to upregulation of TG sources.^17^ Obesity could result in liver inflammation due to steatosis or increased hepatocyte stress attributable to hepatocyte autonomic inflammation (autocrine effect), which may activate inflammatory pathways. Pro‐inflammatory cytokines are the key stimulating components in pathological process of fat accumulation in hepatocytes and liver steatosis.^18^ After adipose tissue causes metabolic inflammation which progresses to the liver with time, it means that the liver does not the main factor responsible for the initial development of metabolic inflammation, but serves as a contributor to metabolic inflammation after its establishment.^19^

The Kupffer cells (hepatic cells similar to macrophages) are also activated through local secretion of cytokine, which has deleterious effects on liver function inflammation and insulin resistance (IR),^20^ and IR is a key factor in progression of NAFLD.^21^ Obesity is also accompanied by hormones derived from fat tissue, while CD4^+^ T cells from the blood contribute to NAFLD.^22^, ^23^ Besides, obesity can increase free fatty acid (FFA) circulation in tissues. They are intricately involved in pathogenesis of pro‐inflammatory occurring in adipocytes, vascular endothelial cells, and myeloid derivatives cells. These physiological events mediated by obesity lead to development of systemic inflammation.^24^ Chronic inflammation triggered by obesity may further increase the risk of liver cancer.^25^

## 2 THE RELATIONSHIP BETWEEN AT INFLAMMATION AND HEPATIC STEATOSIS

AT plays an active and crucial role during progression of liver steatosis and chronic low‐grade inflammation, it also takes a pivotal position in obesity and metabolic disorders.^16^ It has been depicted for one of the body's largest endocrine organs and is an active tissue that has critical functions in cellular responses and metabolic homeostasis, not just an inert tissue that stores energy.^26^ Excess adiposity and adipocyte dysfunction induce a significant amount of AT secretion factors (known as adipokines) dysregulation, which may exert their effects results in the development of NAFLD by altering glucose and lipid homeostasis concomitantly with inflammatory responses^27^ (Figure 1).

Under normal energy balance conditions, adipocytes and immune cells exert an additive or synergistic approach to strictly regulate the storage or consumption of energy. However, accompanied by radical changes in composition of immune cells in obese AT, alongside abnormal production of cytokines and chemokines, increased expression of various inflammatory receptors/ligands, and activation pathways of inflammatory signals, these factors promote AT inflammation.^28^ In obese individuals, hepatic steatosis is intensified by the rising supply of FFA from AT to the liver.^29^ Since excessive FFAs directly access the liver through portal circulation, increased concentrations of FFAs in the liver can cause synthesis of augmenting lipid, and gluconeogenesis concomitant increase in IR.^30^ The levels of interleukin‐6 (IL‐6) in AT is about 100 times higher than that in plasma, and high mobility group box 1 (HMGB1) secretion in AT of patients with obesity is twice as higher than in normal‐weight individuals. Both cause macrophages to secrete pro‐inflammatory cytokines, and HMGB1 has been identified as an inflammation alarmin.^31^ AT affects many other tissues including the liver, heart, and skeletal muscle by augmented secretion of FFAs and many pro‐inflammatory as well as anti‐inflammatory factors, thus plays a central role in the pathogenesis of dyslipidemia, IR, and NAFLD.^32^

AT is the predominant source of systemic inflammatory response, because it boosts the expression of a variety of adipokines, such as chemokines and pro‐inflammatory cytokines.^33^ Macrophages in the stromal vascular part of the AT could be the main cell type involved in generation of IL‐6 and TNF‐α from AT, due to the recruitment of M1 macrophages, which are activated in a classical manner, resulting in increased expression of inflammatory cytokines.^34^ In fact, the number of macrophages is 32% more in white AT than in brown AT.^35^ The level of MCP‐1 in white AT is positively correlated with adiposity. Since the receptor for MCP‐1 and CCR2 are expressed on both AT macrophages and peripheral blood mononuclear cells,^36^ this indicates that chronic low‐grade inflammation upregulated in obese AT could also “transfer” to other tissues through the appearance of active inflammatory mediators in the bloodstream, which occurs by migration from blood mononuclear cells to visceral AT, and then differentiate into macrophages.^37^

Studies have shown that AT transplantation from obese mice can rapidly contribute to the accumulation of circulating neutrophil and hepatocyte infiltration. In addition, NASH model experiment revealed that transplanting AT rich in CD11c^+^ from obese mice, resulted in potentiation of liver macrophage accumulation and liver damage.^38^ Obesity is the primary consequence of the buildup of CD11c+ macrophages in AT, it generates more neutrophil regulatory and stimulating proteins, which in turn concomitantly upregulated neutrophil levels in the blood circulation.^39^ The resulting hepatic neutrophil infiltration could be more efficient in increased macrophage accumulation and is the causative pathway for development of NASH.

The death of adipocytes may be the main factor responsible for obesity. Obesity is accompanied by AT remodeling, which changes the number and size of fat cells.^40^ The size of adipocytes profoundly affects their ability to store lipids, leading to functional changes (such as mitochondrial metabolism impairment) and ultimately to cellular dysfunction as well as death.^41^ The death of adipocytes can also have deleterious effect on local inflammation while whole‐body IR, further contributes to obesity.^42^

The liver identifies nutrient stimulation and has the potential of being very efficient and rapidly produces various nutrients as well as biologically active substances as a key component of energy homeostasis in the whole body.^43^ Obesity triggers ectopic accumulation of lipids in the liver, as an underlying cause of hepatic steatosis. Furthermore, systemic IR reduces the energy storage capacity of AT and provokes the progression of inflammatory state in AT.^44^ Obesity increasingly induces the infiltration of immune cell subtypes and secretion of adipokines, by which a vicious cycle is established between AT inflammation and hepatic steatosis.^45^

## 3 THE RELATIONSHIP BETWEEN INFLAMMATORY FACTORS IN THE BLOOD AND HEPATIC STEATOSIS

**Figure 2:**
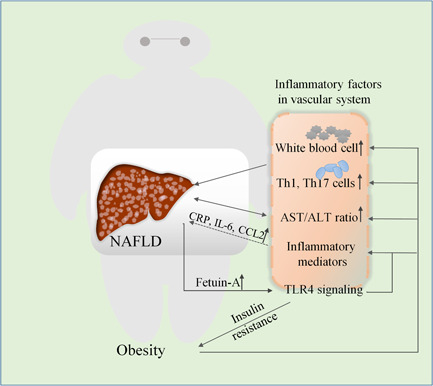
Relationship between obesity‐related vascular inflammatory factors and NAFLD. Blood levels of immune mediators (such as CRP, IL‐6, and CCL2), white blood cell, and TH1, TH17 cell numbers increase with the severity of obesity, contributing to the development of NAFLD. Excessive secretion of Fetuin‐A from liver in NAFLD patients activates TLR4 signaling, which impairs insulin receptor signaling and promotes the release of pro‐inflammatory cytokines. Serum factors such as the AST/ALT or AA/EPA ratio can reflect the progression and severity of obesity and NAFLD. AA/EPA, arachidonic acid/eicosapentaenoic acid; AST/ALT, aspartate aminotransferase/alanine aminotransferase; CRP, C‐reactive protein (CRP); CCL2, CC chemokine ligand 2; IL‐6, interleukin 6; NAFLD, nonalcoholic fatty liver disease; TLR4, Toll‐like receptor4

Obesity is an important preventable cause of death and is a critical risk factor for cardiovascular diseases.^46^ Diagnosis of NAFLD helps in early detection of other serious obesity‐related complications, such as cardiovascular disease. Analysis of hematological parameters of patients with metabolic syndrome revealed that the severity of the patients was significantly correlated positively with related parameters, including neutrophil count, white blood cell count, total lymphocyte count, and red blood cell count. Increased levels of white blood cell count may result in low levels of chronic inflammatory damage to endothelial function, which causes functional impairment in the production of nitric oxide and prostacyclin, which further aggravates vasoconstriction and hypertension.^47^ Studies have showed that white blood cells, neutrophil counts, lymphocyte counts, platelet counts, and systemic immune‐inflammatory index are severely affected by the body mass index (BMI) and are positively correlated with BMI.^48^ After weight loss among patients with obesity, circulating levels of these obesity‐related inflammatory mediators such as serum amyloid A (SAA), C‐reactive protein (CRP), pro‐inflammatory cytokines including IL‐6, and chemokines such as CCL2 (CC chemokine ligand 2)/MCP‐1, IL‐8, and CCL5/RANTES (modulation of activation and normal T cell expressed and secreted) decreased.^49^ Several previous studies suggest that pro‐inflammatory cytokines, especially TNF‐α and IL‐6, play an important role in the phase of NASH^50^ (Figure 2). TNF‐α and IL‐6 level are increased in the liver and blood of patients with NASH, while suppression of these cytokines improves NAFLD in rodents.^51^

As afore‐mentioned, there is a positive correlation between circulating CD4^+^T cells and elevated BMI or obesity.^52^ According to the biosynthesis of specific cytokines, CD4^+^T cells are allocated to four different categories: Th1 cells (characteristic cytokines: interferon‐γ), Th2 cells (characteristic cytokines: IL‐4 and IL‐13), Th17 (characteristic cytokine: IL‐17), and regulatory T (Treg) cells (characterized cytokines: IL‐10).^53^ Generally, Th1 and Th17 cells stimulating M1 macrophages to activate pro‐inflammatory responses, while Th2 and Treg cells are recognized as anti‐inflammatory responses.^54^ In patients with obesity, Th1 and Th17 cells are activated while Th2 and Treg cells are downregulated to exhibits feature of a pro‐inflammatory state.^55^ Furthermore, in nonalcoholic fatty liver experiments, it was found that reducing Th1 and Th17 cells and increasing Th2 and Treg cells in vivo to abolished the immune response, this property is vulnerable to developed for ameliorates NAFLD.^56^

Fetuin‐A (Feta) is a 64 kDa glycoprotein secreted from the liver and AT.^57^ It is involved in accumulation of hepatocyte triacylglycerol and fibrosis or liver inflammation.^58^ It is observed in serum of fatty liver patients at high concentrations and can block insulin‐stimulated glucose transporter 4 translocation and activation of protein kinase B, along with impaired insulin receptor signaling.^59^, ^60^ The study further revealed that FetA forms a ternary complex with nonesterified fatty acids (NEFAs) and TLR4 that activates TLR4 signaling^61^; the accumulation of macrophages then infiltrates AT and subsequently converts to classically activated M1 subtype, thus plays a pivotal role in promoting the release of pro‐inflammatory cytokines.^62^

Clinically, it is important to identify patients at increased metabolic risk and cardiovascular complications based on inflammatory factors in the blood. Limited availability of human metabolic tissue samples (intestine, fat, and liver), hinders clinical studies with control groups, while peripheral blood monocytes are easily harvested from blood.^63^ NAFLD has been considered as an active source of hypertransaminaemia in children and adolescents,^64^ and a reasonable threshold needs to be set to detect the presence and/or severity of liver steatosis through elevated alanine aminotransferase (ALT).^65^ A variety of metabolic disorders originating from obesity are concomitantly upregulated with ALT,^66^ specifically NAFLD is usually a prerequisite for unexplained mild ALT elevation,^67^ in this regard, assessing the aspartate aminotransferase (AST) value is of great importance because elevated AST/ALT ratio is prominently associated with a progressive and more severe condition, such as fibrotic NASH (Figure 2). High levels of gamma‐glutamyl transpeptidase in serum are also risk factors for advanced fibrosis in NAFLD.^68^ Besides, serum bile acid levels decrease in early stages of NAFLD and increase as it progresses to fibrosis.^69^ Arachidonic acid (AA) is involved in the progression of inflammation, while eicosapentaenoic acid (EPA) has antioxidant and anti‐inflammatory effects. The imbalance of AA/EPA ratios in the whole blood and erythrocyte membrane phospholipids may serve as a stimulators for production of more fatty acids, leading to development of different metabolic disorders, including cardiovascular disease and NAFLD.^70^

## 4 THE RELATIONSHIP BETWEEN INTESTINAL INFLAMMATION AND HEPATIC STEATOSIS

**Figure 3:**
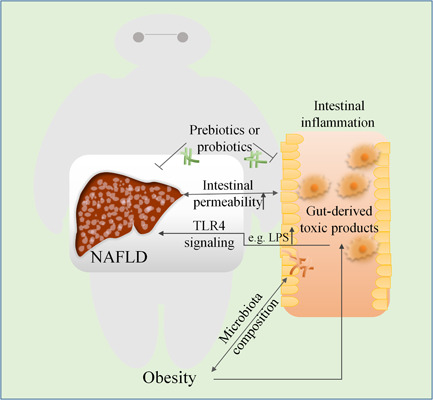
The relationship between obesity‐induced intestinal inflammation and NAFLD. Obesity has been linked to changes in the composition of the human intestinal microbiota. Mechanistically, it activates inflammatory pathways in the gut, thereby changing the intestinal microbiota composition, this process can affect obesity and pro‐inflammatory status. Chronic inflammation in the intestines induces liver toxicity due to gut‐derived toxic products, such as LPS. These products trigger NAFLD by activating the pathway of immune and inflammatory responses, as well as other downstream TLR4, IRAK, JNK, and IKK complexes. Inflammation increases the severity of liver inflammation by enhancing intestinal permeability. Liver damage in nonalcoholic steatohepatitis further increases intestinal permeability and exacerbates intestinal inflammation. The use of prebiotics (which promote the growth of good intestinal flora) and probiotics (living microorganisms) in the gut flora can be beneficial for ameliorating both NAFLD and intestinal inflammation. IKK, I‐kappa B kinase; IRAK, interleukin‐1 receptor associates kinase; JNK, C‐Jun N‐terminal kinase; LPS, lipopolysaccharide; NAFLD, nonalcoholic fatty liver disease; TLR4, toll‐like receptor 4

NAFLD is a predominant health problem associated with the trend toward an unhealthy diet. Dietary fat impairs human health by control of intestinal microbiota composition and low‐grade systemic inflammation.^71^ Monounsaturated fatty acids, saturated fatty acids, polyunsaturated fatty acids, and conjugated linoleic fatty acids activate/inhibit intestinal microorganisms that originate from the same important immune system, thus play a regulating role in obesity and pro‐inflammation. Inflammation increases the permeability of the intestine accompanied by reduction of thickness of the intestinal mucus layer, resulting in severity of inflammation, promotion of a vicious cycle of obesity, increasing intestinal permeability, and inflammation.^72^ These changes increase liver inflammation and IR accomplished by the activation of TLR4. Further, a tight connection has been established between the liver and the intestine, whereby, the liver receives approximately 70% of blood supply directly from the intestine through the portal vein. Hence, it is greatly exposed to gut‐derived toxic products, such as bacteria and bacterial derivatives. This crosstalk between the liver and intestine is well‐referred to as the “gut‐liver axis” and is implicated in the regulation of liver pathologies, including NAFLD^73^ (Figure 3).

Although studies have shown that AT contributes to the onset of obesity‐related inflammation, it has recently been discovered that intestinal immune homeostasis and impaired mucosal barriers are also involved in obesity‐related inflammation.^74^ Obesity and inflammatory bowel disease (IBD) are both systemic inflammatory diseases, which are chronic risk factors associated with a continuously increased risk of thrombosis. They are also involved in arterial and venous thrombosis linked to similar prethrombotic mechanisms,^75^ and act as the major determinant of NAFLD progression. Strong intestinal inflammatory phenotypes in NAFLD cases and coexistence of liver inflammation in inflammatory bowel disease have also been reported.^76^, ^77^

Intestinal flora has critical roles in various stages of occurrence and development of NAFLD, and potential explanations include excessive bacterial growth, intestinal leakage, increased endotoxin absorption, and inflammation.^78^ Particularly in the obese state, the intestinal microbiota contributes to energy intake. Preliminary evidence from animal studies indicates that it is sufficient enough to affect body composition. Germ‐free mice have less body fat and body weight than wild‐type mice, even under high‐fat and high‐sugar diet. Symbiotic bacteria in the intestine can also confer a beneficial influence on intestinal development and maturation as well as to systemic immune system.^79^

When microorganisms from infants of obese mothers are transplanted into germ‐free mice, it results in continuous impairment of their innate immune cell function, and the composition of the intestinal microbiome changes in infants from obese mothers. This leads to increased intestinal permeability, reduction of macrophage phagocytosis activity and bacterial translocation to the liver, which plays a leading role in increased hepatic inflammatory response and triggers NAFLD and weight gain in humanized germ‐free mice.^80^ In contrast, a high‐fat diet fed murine model of NASH revealed that hepatic injury in nonalcoholic steatohepatitis, similarly contributes to altered intestinal permeability.^81^

It has also been evidenced that intestinal microbiota is associated with intestinal permeability. The gut microbiota activates immune signal transduction pathways concomitantly thus affecting intestinal permeability, which leads to chronic low‐grade inflammation that increases the risk of obesity‐related co‐morbidities.^82^ Whereas, once the intestinal bacteria and endotoxins cross the portal vein and/or lymphatic system, they can easily be trafficked to other tissues and organs. This triggers a cascade of reactions accomplished by inflammatory mediators, leading to systemic inflammatory reactions that further damage the intestine barrier.^83^ This greatly increases the risk of the liver being exposed to pathogen‐associated molecular patterns (such as lipopolysaccharide/endotoxin) and/or other products of intestinal tissue damage (such as damage‐associated molecular patterns).^84^ The key representative of lipopolysaccharide (LPS) is that gram‐negative pathogenic strains are characterized by pattern recognition receptors and play an important role in activating the pathway of immune and inflammatory responses. Alongside triggering downstream signalings, such as TLR4, IL‐1 receptor associates kinase, JNK, and I‐kappa B kinase complexes,^85^ it causes persistent low‐grade inflammatory responses. The responses of monocytes, macrophages, and neutrophils, as well as nonimmune cells, such as adipocytes and endothelial cells, to lipopolysaccharide, may correlate with obesity‐related diseases, such as NAFLD.

The use of prebiotics (a substance beneficial for the growth of good intestinal flora) and probiotics (living microorganisms) in the intestinal flora have great contribution towards weight loss.^86^ Extensive studies on lactobacillus rhamnose in NAFLD, have revealed that it can modulate the intestinal flora, reverse small intestinal barrier, reduce hepatitis, improve lipid metabolism, cause progressive decrease in weight and liver fibrosis.^87^

## 5 THE RELATIONSHIP BETWEEN SKELETAL MUSCLE INFLAMMATION AND HEPATIC STEATOSIS

**Figure 4:**
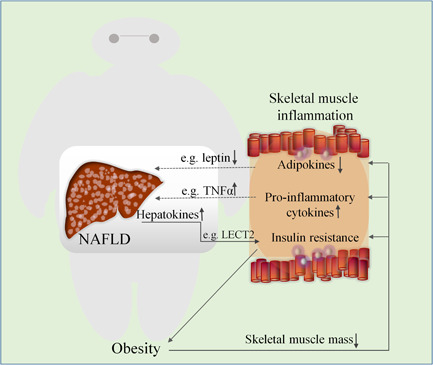
The relationship between obesity‐induced skeletal muscle inflammation and NAFLD. Obesity leads to ectopic lipid accumulation in skeletal muscle and triggers inflammatory response by decreasing skeletal muscle mass, aggravate insulin resistance, upregulating pro‐inflammatory cytokines (e.g., TNF‐α), and downregulating adipokines (e.g., leptin, and adiponectin), all of which contribute to the onset and progression of NAFLD. Inflammation of skeletal muscle decreases insulin sensitivity, thereby modifying obesity and the development of associated NAFLD; conversely, NAFLD as well as alters the factors (e.g., LECT2 and hepassocin) secreted by the liver, further promotes skeletal muscle insulin resistance and exacerbate the inflammatory response in skeletal muscle. LECT2, leukocyte cell‐derived chemotaxin 2; NAFLD, nonalcoholic fatty liver disease; TNF‐α, tumor necrosis factor‐α

Skeletal muscle plays a crucial role in regulation of insulin‐mediated glucose uptake. It is highly sensitive to insulin and is responsible for a large percentage of body weight, so fat accumulation and the subsequent impaired insulin sensitivity may play a vital role in NAFLD.^88^ Progression of obesity leads to increased inflammation in skeletal muscle. AT inflammation may enhance the action of IR via endocrine effects of inflammatory molecules secreted by adipokines on insulin sensitivity in skeletal muscle (SM).^89^ Besides, preadipocyte/adipocyte dysfunction accelerates AT fat spillover into the SM and liver, resulting in ectopic fat deposition and IR in these tissues.^90^ Abnormal body components caused by obesity‐related increase in visceral fat and decrease in skeletal muscle mass, known as “sarcopenia,” provokes IR, resulting in the onset and progression of NAFLD mediated by abnormal glucose and lipid metabolism,^91^ which is a complex process involving pathophysiology of chronic liver disease^92^ (Figure 4). The degree of skeletal muscle reduction is also responsible for the prognosis of patients undergoing liver transplantation.^93^

Coinciding with these inflammation‐related changes, shifts in fatty acid metabolism can contribute to the accumulation of fatty acid intermediates in the liver and skeletal muscle. These can act as ligands for extensive activation of inflammatory pathways in kupffer cells and AT macrophages accomplished by the toll‐like receptor 2 and 4 (TLR2/TLR4) signaling pathway.^94^ At the same time, skeletal muscle myocytes trigger the expression of various cytokines such as IL‐6, and IL‐15, as well as other molecules such as fibroblast growth factor 21 (FGF21), myonectin, and myostatin (known as myokines).^95^ They affect myocardial cells and immune cells, locally through autocrine or paracrine effects. They also control and promote other cells such as adipocytes and liver cells through endocrine effects, which in turn display a marked reduction in adverse effects of adipokines and confers a beneficial influence on lipid metabolism, glucose and inflammation.^96^

In patients with obesity, in terms of the size and number distribution of adipocytes in the intramuscular fat stores, there was the vast majority of infiltrated by pro‐inflammatory macrophages. Adipocytes and macrophages are the main sources that elicit a marked upregulation of synthesis and secretion of adipokines (adiponectin and leptin) as well as pro‐inflammatory cytokines (IL‐1, TNF‐α, and IL‐6).^97^ IL‐1 can synergize with TNF‐α and IL‐6, to exert a negative impact on biological function of adipocytes.^98^ It has also been found that a positive relationship exists between the presence of NASH and the expression of CD3 in skeletal muscle, which is associated with liver fibrosis through biopsy assessment.^99^

Previous studies indicate that NAFLD confers a harmful effect on the factors secreted by the liver, including lipids, hepatokines, and noncoding RNAs.^100^, ^101^ These factors (e.g., leukocyte cell‐derived chemotaxin 2) broadly act on distant tissues such as muscle tissues through autocrine and paracrine signal transmission and are transported through systemic circulation.^102^ The balance between pro‐inflammatory and anti‐inflammatory cytokines is also a contributing factor during the development of muscle IR. Moreover, it has also been found that alongside increased expression of hepassocin due to liver steatosis, it further promotes the development of skeletal muscle IR associated with disruption of this balance,^103^ indicating that NAFLD in turn exacerbates the inflammatory response in skeletal muscle.

## 6 THE RELATIONSHIP BETWEEN BRAIN TISSUE INFLAMMATION AND HEPATIC STEATOSIS

The brain has a crucial role in energy intake and expenditure, among which the hypothalamus is essential for the regulation of body weight and food intake.^104^ Human genome‐wide association studies have identified some neuronal genes that affect obesity, especially genes involved in the regulation of energy balance or appetite in the hypothalamus.^105^ When animals are fed a high‐fat diet, inflammatory changes can be detected in the brains,^106^ the inducible nitric oxide synthase (iNOS) is activated, which enhances the proliferation of macrophages in the hypothalamic arcuate nucleus.^107^ Meanwhile, the production of prostaglandin E2 and reactive oxygen species in the cerebral cortex is increased, as well as upregulation of NF‐κB signaling.^108^ Some canonical pro‐inflammatory cytokines, TNF‐α, IL‐1β, and IL ‐6 are the largest gene types whose expression levels are altered in the hypothalamus.^109^ It also affects the central nervous system through PKC‐θ activation, which has deleterious effects on hypothalamic insulin activity in mice and rats.^110^ Endoplasmic reticulum (ER) stress can also cause inflammation and plays a vital role in the progression of NAFLD,^111^ particularly when it occurs in the brain.^112^ Following focal inflammatory lesions in the brain, enzyme markers of liver tissue damage in serum (AST/ALT) are found to increased,^113^ a large amount of LPS is also transferred from the brain to the circulatory system, stimulating the TLR4‐dependent signaling pathway.^114^

**Figure 5:**
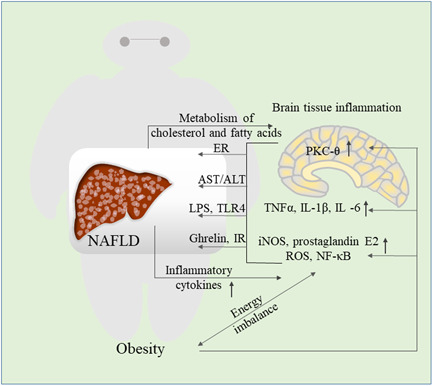
The relationship between obesity‐induced brain tissue inflammation and NAFLD. Overnutrition caused by obesity activates NF‐κB, pro‐inflammatory cytokines, PKC‐θ and other signaling pathways in the brain. Following inflammation of the brain tissue, endoplasmic reticulum stress occurs, AST/ALT increases, and LPS stimulates TLR4 signals, ghrelin induces insulin resistance and promotes liver lipid accumulation. The progress of NAFLD is also accompanied by an increased inflammatory cytokines profile in the brain, at the same time, the metabolism of cholesterol and fatty acids in the brain is unbalanced, which further aggravates the brain tissue inflammation. ALT, alanine aminotransferase; AST, aspartate aminotransferase; ER, endoplasmic reticulum; iNOS, inducible nitric oxide synthase; IR, insulin resistance; LPS, lipopolysaccharide; NAFLD, nonalcoholic fatty liver disease; NF‐κB, nuclear factor‐κB; PKC‐θ, protein kinase C‐θ; ROS, reactive oxygen species

Significant liver inflammation can be observed in the progress of NAFLD, accompanied by an increased inflammatory cytokines profile, numbers of activated microglial cells, and neurodegeneration in the brain.^115^ The liver communicates with the brain including the hypothalamus through sympathetic and parasympathetic, the hypothalamus can send signals to the liver via a network of neurons to regulate VLDL production and lipid content.^116^ The imbalance of interorgan crosstalk between the brain and liver may be an important factor of liver TG retention and steatosis in the development of NAFLD^117^ (Figure 5). In addition, the homologous receptors of intestinal hormones (such as peptide YY, GLP‐1, and ghrelin) are also commonly found in the central nervous system (CNS) and can regulate eating behavior,^118^ the acylated ghrelin can induce IR and promote liver lipid accumulation via the hypothalamus.^119^ Generally, the production of vascular endothelial endogenous extracellular vesicles (EVs) is the basis of CNS‐liver inflammatory signaling,^120^ NAFLD‐related CNS is more manifested as neuroinflammation, cerebrovascular alteration and cerebral IR.^121^ NAFLD imbalances the metabolism of cholesterol and fatty acids in the brain, which is related to brain inflammation.^122^ Under certain circumstances, inflammation attenuated in the hypothalamus can reduce adiposity, steatosis, gluconeogenesis and restore leptin sensitivity, result in the amelioration of IR in the liver.^123^

## 7 INFLAMMATION‐RELATED FACTORS AS TARGETS FOR IMPROVING LIVER PATHOLOGY

### 7.1 High mobility group box 1

During the development of steatosis in the liver, from mild inflammation to various degrees of fibrosis or even liver cancer,^124^ HMGB1, which is synthesized by innate immune cells is involved in maintaining inflammation and immune responses and is a contributing factor in this signaling pathway.^125^ The secretion of HMGB1 boosts infiltration of more inflammatory cells and markedly accelerates the activation of innate immune cells in the liver,^126^ which is a causative factor that prolongs chronic inflammatory process in the liver of obese subjects.^127^ Studies have found that the inflammatory response significantly decreases in NAFLD when inhibiting HMGB1.^128^, ^129^ This attenuates the inflammatory response and IR through TLR4 and receptor for advanced glycation end products signaling, which ultimately protects it from NAFLD.^130^ Its expression may be an early indicator of development of obesity and can be implemented as a therapeutic agent to prevent NAFLD or obesity‐related inflammation.

### 7.2 Peroxisome proliferator activated receptor‐α

In hepatocytes, the liver cell type with the highest peroxisome proliferator activated receptor‐alpha (PPARα) expression has critical functions in lipid metabolism, gluconeogenesis, and amino acid metabolism.^131^ PPARα also has a crucial role in the regulation of the inflammatory response in the liver,^132^ which is achieved by reducing the activation and infiltration of inflammatory cells in the liver. PPARα can reduce obesity‐induced hepatitis through reducing fatty liver (which is closely related to elevated inflammatory states), by directly regulating the expression of inflammatory genes or by inhibiting inflammation of AT,^133^ and plays a substantial role during the progression of obesity complicated with NAFLD.

### 7.3 Fibroblast growth factor 21

FGF21 is mainly secreted from tissues with high metabolic activity (such as liver, muscle, pancreas, and AT) and functions in many tissues.^134^ The major pathogenic mechanism in the development of NAFLD is obesity, dyslipidemia, and insulin insensitivity, which are improved by FGF21.^135^ However, its deficiency is associated with significant deterioration of liver steatosis, apoptosis, fibrosis, inflammation, and the progression of severe NASH.^136^ Impressively, under conditions of FGF21 treatment, it can reduce or eliminate the methionine‐choline deficient diet‐induced progression to NASH.^137^ The activation of Th17 cells and elevated IL17A levels often trigger NASH.^138^ Notably, the anti‐inflammatory activity of FGF21 in NASH is related to its suppression of IL17A production.^139^ The FGF21 is also positively involved in the regulation of adiponectin, which depends on adiponectin to exert a systemic effect on energy metabolism and insulin sensitivity.^140^ Further, adiponectin inhibits Th17 cells by the direct action of Sirtuin1 (SIRT1)/PPARγ pathway.^141^ Besides, FGF21 enhances fatty acid oxidation in hepatocyte by upregulating long‐chain acyl CoA synthetases/fatty acid transport proteins expression alongside promoting mitochondrial β‐oxidation of fatty acids.^142^

Considering the impact of obesity on inflammation or NAFLD, special attention should be paid to the treatment of inflammation or NAFLD, especially in patients with obesity. Studies have showed that using tocilizumab, a humanized anti‐IL‐6 receptor antibodies can inhibit lipolipolysis and low‐density lipoprotein (LDL) receptor expression in the liver, which in turn induces total cholesterol (TC), while triglycerides ester (TG) and LDL levels are abnormally upregulated.^143^ Besides, anti‐TNF‐α may also worsen the lipid profile and similarly cause an increase of TC and LDL,^144^ further, this treatment can also result in significantly elevated TG.^145^

## 8 CONCLUSION AND FUTURE PROSPECTS

**Figure 6:**
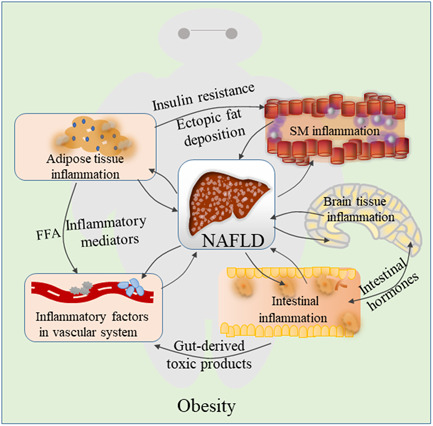
The role of obesity‐induced inflammation in nonalcoholic fatty liver disease (NAFLD). Obesity plays a role in the development of associated NAFLD by affecting several inflammatory reactions via adipose tissue, vascular, intestinal, skeletal muscle and brain, this process is associated with adipose tissue inflammation, inflammatory factors in the blood, intestinal inflammation, skeletal muscle inflammation and brain tissue inflammation. Obesity and NAFLD are the net effects of these changes. Once NAFLD has been established, it contributes to systemic inflammation, and the low‐grade inflammation is sustained during NAFLD leading to impaired resolution of inflammation in obesity, which subsequently can in turn exacerbates the severity of obesity. SM, skeletal muscle

Obesity is accompanied by inflammatory reactions in various body tissues, mainly liver tissue, AT, the intestine tissues, skeletal muscle, blood and brain. High level of inflammatory factors leads to excessive accumulation of lipids in the liver (steatosis), which in turn triggers lipotoxicity, hepatocyte cell death, liver inflammation, fibrosis, and pathological angiogenesis. Understanding the impact of obesity on inflammatory response and its interactions in the metabolism as well as cellular signaling systems is critical to prevention or treatment of NAFLD (Figure 6). Several studies have shown that controlling inflammation may be an effective approach to treat NAFLD. Analyzing the relationship between obesity‐induced inflammation and NAFLD is currently a challenging issue, the attractive therapeutic targets or diagnostic markers may be found from the inflammatory response. Further research is to explore how inflammatory response associates with energy metabolism in NAFLD. Moreover, studies should focus on understanding relationship between inflammatory response and obesity, and how this impacts on physiology and NAFLD. This will lead to identification of appropriate treatment approaches for obesity‐associated diseases.

## CONFLICT OF INTERESTS

The authors declare that there are no conflict of interests.

## AUTHOR CONTRIBUTIONS

Yunfei Luo and Hui Lin wrote the manuscript.

## Data Availability

The data that support the findings of this study are available from the corresponding author upon reasonable request.
